# Age-Dependent Prevalence of Nasopharyngeal Carriage of *Streptococcus pneumoniae* before Conjugate Vaccine Introduction: A Prediction Model Based on a Meta-Analysis

**DOI:** 10.1371/journal.pone.0086136

**Published:** 2014-01-23

**Authors:** Olivier Le Polain de Waroux, Stefan Flasche, David Prieto-Merino, W. John Edmunds

**Affiliations:** 1 Department of Infectious Disease Epidemiology, London School of Hygiene and Tropical Medicine, London, United Kingdom; 2 Department of Medical Statistics, London School of Hygiene and Tropical Medicine, London, United Kingdom; The University of Tokyo, Japan

## Abstract

**Introduction:**

Data on the prevalence of nasopharyngeal carriage of *S.pneumoniae* in all age groups are important to help predict the impact of introducing pneumococcal conjugate vaccines (PCV) into routine infant immunization, given the important indirect effect of the vaccine. Yet most carriage studies are limited to children under five years of age. We here explore the association between carriage prevalence and serotype distribution in children aged ≥5 years and in adults compared to children.

**Methods:**

We conducted a systematic review of studies providing carriage estimates across age groups in healthy populations not previously exposed to PCV, using MEDLINE and Embase. We used Bayesian linear meta-regression models to predict the overall carriage prevalence as well as the prevalence and distribution of vaccine and nonvaccine type (VT and NVT) serotypes in older age groups as a function of that in <5 y olds.

**Results:**

Twenty-nine studies compromising of 20,391 individuals were included in the analysis. In all studies nasopharyngeal carriage decreased with increasing age. We found a strong positive linear association between the carriage prevalence in pre-school childen (<5 y) and both that in school aged children (5–17 y olds) and in adults. The proportion of VT serotypes isolated from carriers was consistently lower in older age groups and on average about 73% that of children <5 y among 5–17 y olds and adults respectively. We provide a prediction model to infer the carriage prevalence and serotype distribution in 5–17 y olds and adults as a function of that in children <5 years of age.

**Conclusion:**

Such predictions are helpful for assessing the potential population-wide effects of vaccination programmes, e.g. via transmission models, and thus assist in the design of future pneumococcal conjugate vaccination strategies.

## Introduction

Colonization of the nasopharynx by *Streptococcus pneumoniae* is the reservoir for *S.pneumoniae* transmission and a prerequisite for pneumococcal disease [1]. Pneumococcal conjugate vaccines (PCV) reduce nasopharyngeal carriage of serotypes included in the vaccine by conferring capsular-specific immunity. Experience from countries where conjugate vaccines have been introduced has shown rapid and sustained carriage reduction of vaccine serotypes (VT) following vaccination. Those trends have been observed not only among vaccinated children but more widely across all age groups through a strong herd immunity effect [2], [3]. Despite evidence of almost complete serotype replacement in many settings, whereby non-vaccine serotypes (NVT) colonise the space left vacant by vaccine type (VT) serotypes [4], pneumococcal conjugate vaccination programmes have led to a substantial reduction in severe disease due to the lower propensity of replacing serotypes to cause disease [4], [5].

Ten- and thirteen-valent pneumococcal conjugate vaccines (PCV10 and PCV13) are now being introduced into the routine immunization programmes of many developing countries (www.jhsph.edu/ivac/vims.html), where their impact is expected to be high, given the disproportionate burden of pneumococcal disease in such settings [6].

Estimates of the potential impact of routinely introducing pneumococcal conjugate vaccines (PCVs), however, crucially depend on the nasopharyngeal carriage prevalence in the population before the introduction of PCV, the distribution of serotypes (VT and NVT) within the population, including among older children, adults and the elderly, and the propensity of replacing serotypes to cause disease across age groups.

Most carriage surveys are limited to children under five years of age, in whom the disease burden is high and for whom sample size requirements for precision are reasonable given the high carriage prevalence. As a result, nasopharyngeal carriage estimates in other age groups are scarce. However, such estimates are important to help predict the overall population impact of vaccination programmes as well as the specific impact among unvaccinated age groups. Routine infant PCV vaccination has been found to also impact substantially on the elderly in whom the likelihood to develop severe pneumococcal disease as a result of carriage is high [7], [8] and who present the highest overall burden of pneumocccal associated disease in developed countries [9]. Hence this age group has also played an important role in the cost effectiveness considerations of pneumococcal conjugate vaccination [10], [11]. With the proportion of population of >60 years old growing at its fastest pace ever [12], the indirect effect of PCV vaccination programmes may become increasingly important, including in developing countries.

The overall aim of this study was to explore a possible correlation between the prevalence and distribution of *S.pneumoniae* serotypes carried in the nasopharynx of children <5 y and that in older children (5–17 y olds) and adults (≥18 y olds), based on nasopharyngeal carriage surveys, and further establish predictors for carriage prevalence and serotype distribution in adults and older children as a function of the carriage prevalence and serotype distribution in the nasopharynx of children under five years of age.

## Methods

### Search Strategy

We conducted a systematic review to identify articles reporting nasopharyngeal carriage prevalence estimates for different age strata. We used MEDLINE and Embase electronic databases to retrieve articles published between the date of the earliest articles compiled on MEDLINE (1946) or Embase (1947) and 23^rd^ August 2013 (i.e. week 35), and used the following combination of search terms: ‘(pneumonia OR pneumoniae OR pneumococcal OR pneumococcus) AND (carriage OR colonization OR colonisation)’ in the title or the keywords or the abstract. No language restriction was applied.

There is no registered protocol for this systematic review.

Our systematic review and meta-analysis was conducted in accordance with the PRISMA checklist (http://www.prisma-statement.org/statement.htm, see Checklist S1) and MOOSE guidelines [13], which compile guidelines for the reporting of meta-analysis of observational studies.

### Eligibility Criteria

We included studies based on seven main eligibility criteria.

Articles were considered for inclusion if they provided (i) pneumococcal nasopharyngeal carriage prevalence estimates (ii) in a population not previously exposed to PCV, with (iii) nasopharyngeal sampling and transport procedures as well as *S.pneumoniae* culture based on WHO guidelines [14], (iv) where the study was not restricted to specific serotypes or to *S.pneumoniae* with specific patterns of antibiotic sensitivity. Studies were further considered for inclusion if they provided carriage estimates in young children, as well as in older age groups and (vi) in individuals not suffering from any acute respiratory infection or any confirmed pneumococcal disease, and (vii) were not based on particular at risk population groups such as HIV positive individuals.

No design restriction was applied.

### Data Extraction

The articles were screened and reviewed with inclusion criteria appraised in the order described above. When primary data published in a study were also used in subsequent studies, we screened the latter too to find any data that may not have been published in the original paper. For each study meeting the aforementioned inclusion criteria we calculated the prevalence of nasopharyngeal carriage by age group, as well as the prevalence of VT and NVT by age group when provided, for PCV7 and/or PCV10 and/or PCV13, depending on available data. In most studies the group of NVT comprised of NVT serotypes as well as non-typeable (NT) serotypes, while in a few studies estimates for NT by age group were provided separately and were therefore not included in the group of NVT serotypes.

In some studies estimates were provided by age or for smaller age bands, and such estimates were therefore pooled to obtain estimates for the main four age groups considered.

In longitudinal studies where multiple nasopharyngeal swabs were taken for each individual, the number of individuals tested positive was approximated by the age-specific average number of positive swabs over the study period.

In a few studies the actual number of carriers had to be estimated based on reported prevalence estimates and the number of study participants in each age group.

More details on how the data were extracted from the different studies can be found in File S1.

### Analysis

We considered the following age groups: <1 y (infants); <5 y (pre-school children including infants); 5–17 y (school aged children) and ≥18 y (adults). Because age groups were not standardised between studies, the category of <5 y olds included studies reporting estimates in <4 y olds as well as studies reporting estimates in <6 y olds. The category of school-aged children included any age group from between 4 to 6 years up to any age between 10 years and 19 years, and we considered the prevalence among adults to be that in individuals aged at least 15 years and above.

We explored the association between the carriage prevalence and VT or NVT distribution in older age groups and that in young children using Bayesian linear meta-regression analysis. The use of Bayesian over a frequentist approach was motivated by the natural way in which each study’s contribution to the meta-regression is weighted in a Bayesian approach, and also because Bayesian linear regression is the recommended tool by the Cochrane Collaboration to account for uncertainty around both the outcome and the exposure variables in a meta-regression [15].

#### Age-dependent overall carriage prevalence and carriage prediction

We obtained prediction intervals for the carriage prevalence in adults and in 5–17 y olds as a function of that in either <5 y olds or <1 y olds using a Bayesian linear meta- regression model. For the 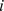 studies included in each analysis,
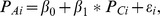



with 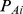 = prevalence in either adults or 5–17 y olds in study 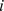, 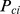 = prevalence in either <5 y olds or <1 y olds in study 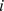 and 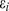 = random error in study 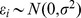.

The true prevalence 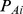 and 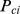 are unknown, however the observed number of carriers in each study (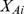 and 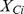) follows a binomial distribution. Hence, based on these and on the sample sizes (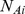
and 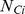) it follows that 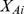 ∼ Binomial (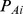, 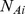) and ∼ Binomial (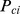, 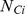).

We provided prior distributions for the parameters of interest 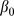, 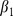 and 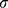 and assigned uniform uninformative priors to 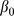 (unif (−1,1)) and 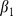 (unif (−5,5)) and to 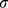 (unif (0,0.4)).

The posterior distributions were obtained through a Markov Chain Monte Carlo (MCMC) Gibbs sampling algorithm, with 100,000 iterations of 2 chains running in parallel, after a burn-in of 5,000 iterations. We retained one in five iterations in the posterior sample to limit autocorrelation. Convergence of the chains was examined visually. We obtained the 95% posterior credible intervals (CrI) for the model regression line as well as the posterior credible intervals for the prediction of individual studies by including the prediction error (which we will refer to as the “prediction interval” in the results section).

We further explored the possible association of socio-demographic and geographic characteristics of the populations studied through Bayesian multivariable meta-regression. The explanatory variables considered for meta-regression included the proportion of children <5 y of age and the % <15 y of age in the country or area in which the study took place based on figures obtained from UN Population data (http://esa.un.org/wpp/), categories of national income level in the country or area in which the study took place, based on estimates from the World Bank (http://data.worldbank.org/), and broad geographical areas or continents in which studies took place. Variables were added one after another in the model and were retained if the 95% posterior probability interval for their coefficient excluded 0.

#### Age-dependent distribution of VT serotypes

We then explored (i) the distribution of the group of VT serotypes among carried serotypes in children <5 y and in older age groups, and the association between such distributions, as well as (ii) the relationship between the prevalence of VT carriage and NVT carriage in adults and in 5–17 y olds compared to children <5 years of age.

For each study providing serotype-specific information we calculated (i) the proportion of VT serotypes (for PCV7 or PCV10 or PCV13) among carriers in children and older age group, and their 95% confidence interval (CI) and (ii) the carriage prevalence of such groups of serotypes, by age category and 95% CI.

We further explored the relationship between (i) the proportion of VT carriers and (ii) the VT and NVT carriage prevalence across age groups using Bayesian linear meta-regression. We used the same uniform priors for 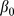, 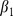 and 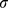 than in the analysis of overall carriage prevalence, as well as the same analytical strategy to obtain posterior estimates.

The code used for the Bayesian linear meta-regression can be found in File S2 and is fully annotated. In addition, File S2 also provides an opportunity for readers to obtain posterior distributions of the carriage prevalence in 5–17 y olds and adults based on study-specific estimates of nasopharyngeal carriage in <5 y olds, making it possible to use this as a carriage prediction tool based on specific data of carriage in <5 y olds.

Analyses were performed using R software and the JAGS package in R (http://mcmc-jags.sourceforge.net/).

## Results

**Figure 1:**
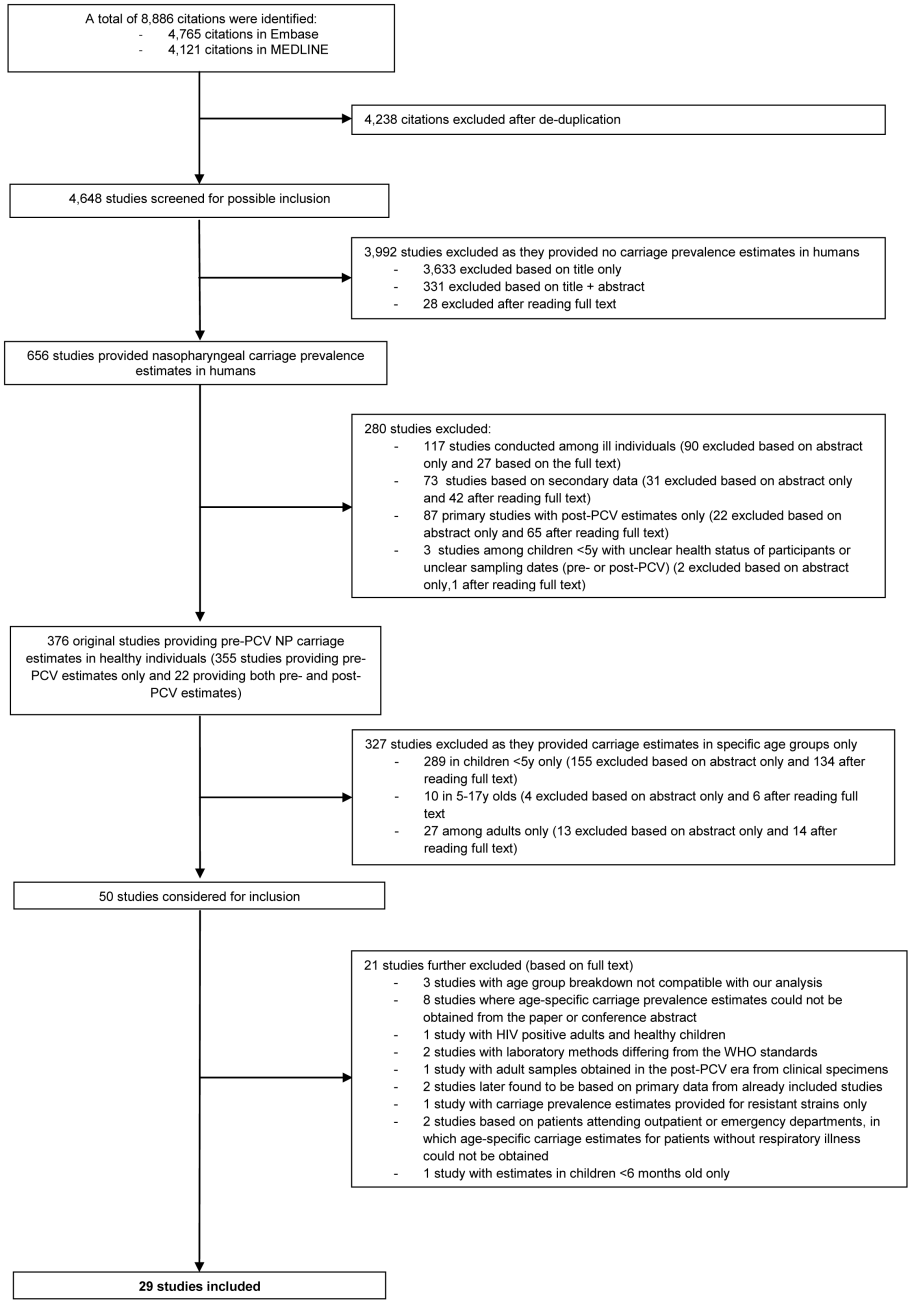
Flow chart of the study selection process.

A total of 8,886 citations were found, which amounted to 4,648 citations after duplicates were excluded. Of those, 376 original studies provided pre-PCV nasopharyngeal carriage estimates in healthy individuals. A flowchart of the number of studies screened and reasons for exclusion is displayed in Figure 1.

A total of 29 studies were included in the meta-analysis, comprising a total of 20,391 individuals, including 7931 children <5 y, 3936 school aged children and 8524 adults.

### Age Dependent Prevalence of Nasopharyngeal Carriage

Seven studies were conducted in Africa, 6 in Asia, 6 in the Middle-East, 5 in Europe, 2 in the North America (Alaska, USA) and 3 in South America. Table 1 displays the main characteristics of the studies included in the final analysis, including details about how individuals were selected in each age group, as well as the estimates of carriage prevalence by age group.

**Table 1:** Studies included in the Bayesian linear meta-regression of the overall carriage prevalence.

			No carriers/total individuals (%), by age group					Study population characteristics by age group			
Authors	Country or region	Setting	<1 y olds	<5 y olds	5–17 y olds	≥18 years	Age groups	<1 y	<5 y	5–17 y	≥18 y
Abdullahi et al. (2008) [17]	Kenya	C	58/98 (59%)	198/349 (57%)	55/213 (55%)	16/302 (5%)	<1 y;<5 y; 5–19 y; ≥20 y	RX	RX	RX	RX
Adetifa IM., et al. (2012) [34]	Nigeria	C	143/193(74%)	375/524 (72%)	63/125(25%)	90/356 (25%)	<1 y;<5 y; 5–14 y; ≥15 y		RX	RX	RX
Bello Gonzales et al. (2010) [35]	Venezuela	C		58/84 (69%)		7/64 (11%)	<5 y; ≥18 y		RX		*M*
Cekmez et al. (2009) [36]	Turkey	H		0/125 (0%)*	25/375 (7%)		<4 y; 4–12 y		RH	RH	
Chen et al. (2007) [37]	Taiwan	C&H		25/94 (27%)	18/196 (9%)	0/137 (0%)	3–4 y; 5–17 y; ≥18 y		SC	SC	HC
Dagan et al. (2000) [38]	Israel	C		59/84 (70%)	71/199 (35%)	27/174 (15%)	<6 y; 6–15 y;≥16 y		RX	RX	RX
Darboe MK., et al. (2007) [39]*	Gambia	C	143/196 (73%)			26/196 (13%)	<1 y; ≥18 y	BC			*M*
Dhakal et al. (2010) [40]	India	H		18/79 (23%)	26/120 (22%)		<4 y; 4–12 y		RH	RH	
Granat et al. (2007) [41]	Bangladesh	C	49/99 (49%)	86/172 (50%)	45/117 (38%)	12/154 (12%)	<1 y;<5 y;5–18 y;≥19 y	BC	F	F	*F*
Greenberg et al. (2004) [42]	Israel	H		147/216 (68%)		33/216(15%)	<5 y; ≥18 y		RH		*M*
Hammitt et al. (2006) [3]	Alaska (USA)	C		377/639 (59%)		275/2115 (13%)	<5 y; ≥18 y		RX		RX
Henriqus Normark et al.(2003) [43]	Sweden	C		246/611 (40%)		2/123 (2%)	1–6 y; 19–59 y		SC		*SC*
Hill PC., et al. (2006) [19]	Gambia	C	141/145(97%)	621/666 (93%)	621/735 (84%)	821/1471 (55%)	<1 y;<5 y;5–14 y; ≥15 y	RX	RX	RX	RX
Hussain et al. (2005) [18]**	UK	C		87/180 (48%)	15/71 (21%)	18/237 (8%)	<5 y; 5–17 y; ≥18 y		RX	RX	RX
Inostroza et al.(1998) [44]	Chile	C&H		10/55 (18%)	5/16 (31%)	2/38 (5%)	<5 y; 5–15 y; ≥18 y		SC	SC	HC
Kaltoft et al. (2008) [45]	Denmark	C		340/584 (58%)		23/109 (21%)	mean 23 m (n = 123) and mean 52 m (n = 461); ≥18 y		SC		*F&SC*
Leino et al. (2008) [46]	Finland	C		15/59 (25%)	4/31 (13%)	4/123 (3%)	mean 4 y, 8 y 35 y		SC	F	*F&SC*
Lloyd-Evans N., et al. (1996) [47]	Gambia	C		323/414 (78%)	188/342 (55%)	18/67 (27%)	<5 y;5–18 y; ≥19 y		RX	F	*F*
Lo et al. (2003) [48]	Taiwan	H		75/360 (21%)	20/118 (17%)		<6 y; 6–14 y		RH	RH	
Mueller et al. (2012) [49]	Burkina Faso	C	43/62 (69%)	81/128 (63%)	57/196 (29%)	28/195 (14%)	<1 y;<5 y; 5–19 y; ≥20 y	RX	RX	RX	RX
Nunes et al. (2013) [50]	South Africa	C	83/123 (69%)			21/123 (17%)	<16 m; ≥18 y	BC			*M*
Parry et al. (2000) [51]	Vietnam	C		192/389 (49%)	212/522 (41%)		<5 y; 5–16 y		RX	RX	
Regev-Yochay et al. (2004) [16]	Israel	H	38/90 (42%)	214/404 (53%)		52/1300 (4%)	<1 y;<7 y;≥18 y	RH	RH		RH
Regev-Yochay et al. (2012) [52]	Occup. Palest Terr.^2^	C	43/90 (48%)	189/379 (50%)		30/376 (8%)	<1 y;<5 y; ≥18 y	RX	RX		*F*
Reichler et al. (1992) [53]	USA	C	16/25 (63%)	107/166 (64%)	10/53 (19%)		<18 m;<5.5 y; 5–10 y	SC	SC	SC	
Reis JN. et al. (2008) [54]	Brazil	C		33/50 (66%)	43/95 (45%)	19/117 (16%)	<5 y; 5–17 y; ≥18 y		RX	RX	RX
Sener et al. (1998) [55]	Turkey	C		71/248 (29%)	87/412 (21%)		<6 y;6–11 y		SC	SC	
Turner et al. (2012) [56]	Thailand^1^	C	188/234 (80%)			57/231 (25%)	<2 y; ≥18 y	BC			*M*
van Gils E. et al (2009) [57]	Netherlands	C	214/319 (67%)			67/300 (22%)	12 m; ≥18 y	BC			*F*

H: Health care setting, C: Community setting ^1^Maela refugee camp at the Thailand-Myanmar border. ^2^Gaza strip. *Data from the study extracted from Darboe et al. [58]. ** Data were extracted from Flasche et al. [20].

RX: random sample from cross sectional survey, RH: random outpatient sample, SC: school cohort (i.e. all children or staff in a particular school/DCC/class), M: mothers, F: family members, BC: birth cohort. Italicised are the adults samples where adults are parents or staff members looking after the young children included.

**Figure 2:**
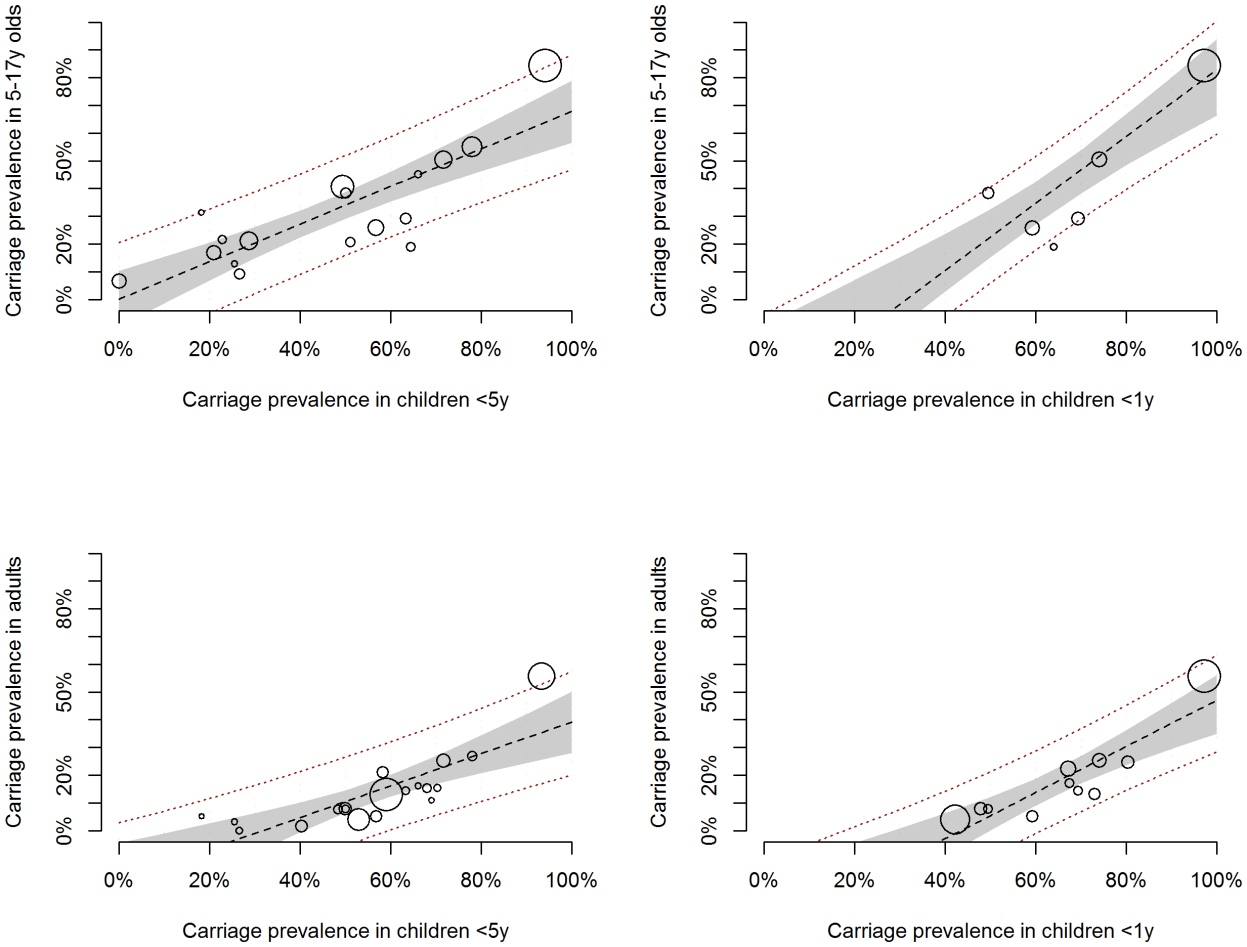
Overall carriage prevalence in older age groups against <5 y olds: scatter plot and fitted model. Each circle corresponds to one study, with the circle size proportional to the study size (i.e. number of individuals contributing to the x and y axis estimates). The lines correspond to the fitted Bayesian linear meta-regression model. The dashed black line shows the median posterior estimate and the grey shaded area the 95% credible interval around the median. The red dotted lines represent the 95% prediction interval.

We found a strong positive correlation between carriage prevalence in younger age groups and that in older age groups. Figure 2 displays a scatter plot of the study specific estimates for the prevalence in adults and in 5–17 y olds as a function of that in either <5 y olds or <1 y olds. The figure also displays the fitted regression line from the Bayesian linear meta-regression model, including the median posterior estimate, the 95% credible interval around the median, as well as the 95% prediction intervals for all four analyses. The model coefficients of all four models can be found in Table 2.

**Table 2:** Model coefficients for each of the Bayesian linear meta-regression models used.

Model	Intercept (β_0_)	Slope (β_1_)
	median (95% CrI)	median (95% CrI)
***Overall carriage prevalence***		
Adults vs. <5 y	−0.18 (−0.31; −0.05)	0.57 (0.35;0.80)
Adults vs. <1 y	−0.36 (−0.49; −0.15)	0.83 (0.52; 1.03)
5–17 y vs. <5 y	−0.00 (−0.10; 0.10)	0.68 (0.48; 0.86)
5–17 y vs. <1 y	−0.38 (−0.49; −0.09)	1.22 (0.79; 1.40)
***VT carriage prevalence***		
Adults vs. <5 y	−0.06 (−0.17; 0.05)	0.34 (0.05; 0.62)
5–17 y vs. <5 y	−0.10 (−0.41; 0.20)	0.73 (0.00; 1.43)
***NVT carriage prevalence***		
Adults vs. <5 y	−0.10 (−0.30; 0.09)	0.76 (0.11; 1.46)
5–17 y vs. <5 y	−0.04 (−0.21; 0.14)	1.27 (0.63; 1.89)
***VT proportion among carriers***		
Adults vs. <5 y	−0.07 (−0.41; 0.33)	0.73 (0.06;1.32)
5–17 y vs. <5 y	−0.03 (−0.41; 0.33)	0.73 (0.10; 1.37)

We explored whether the results from the regression model were confounded by the socio-demographic and geographic characteristics of the study population, but found no evidence that the proportion of children, the national income level or the geographic region of the included studies were associated with the outcome in any of the models considered. In all analyses and for all models considered the values of β1 coefficients were unaffected and the coefficients of the variable explored were centred around zero.

We also explored whether prediction estimates for the adult population differed with studies (n = 11) where a random community or hospital sample of adults was taken compared to studies (n = 8) based on convenience sampling of parents, guardians or day care centre staff looking after children included (see Table 1 for further details). We found no difference in the prediction estimates obtained in subgroup analyses. Figure S1 shows the plot for those analyses.

Specific estimates in older adults were provided in two studies included in the analysis, one in Israel with data in >65 y olds [16] and another in Kenya with data in >50 y olds [17]. Based on the reported data there was no evidence that the carriage prevalence among older adults differed substantially from that in younger adults (Israel: 3.7% in 18–65 y vs. 4.6% in >65 y, p = 0.573 and Kenya: 5.6% in 20–49 y olds vs. 4.6% in ≥50 y olds, p = 0.719).

### Age-dependent Prevalence of VT Carriage and Distribution of VT Serotypes

A total of eleven studies provided estimates of the distribution of VT and NVT serotypes in young children and in older children and/or adults. In all studies included serotyping was performed using standard methods such as latex agglutination and capsular swelling (Quellung reaction).

Estimates from a study in Israel [16] were based on outpatients, including a proportion of patients with upper respiratory tract infections (URTI), as serotype specific estimates could not be obtained for the healthy study participants only. Data on the serotype distribution among participants of two studies [18], [19] were extracted from subsequent manuscripts [20], [21] as the information was unavailable in the original articles. Two studies included in the analysis only provided estimates in <2 y olds rather than <5 y olds. However, as meta-regression coefficients, obtained with and without the inclusion of such studies, were similar we included both studies in the final analysis.

Table 3 provides details of the studies included in the analysis. The proportion of VT serotypes isolated from positive swabs was consistently lower in older age groups compared to children under five, and that of NVT serotypes consistently higher. This finding was consistent across studies.

**Table 3:** Studies included in the Bayesian meta-regression of VT and NVT distribution, with proportion of VT out of positive samples, by age groups.

				<5 y olds	5–17 y olds	Adults	
Studies	Country/Area	PCV valency	Age	VT/all serotypes (%VT)	VT/all serotypes (%VT)	VT/all serotypes (%VT)	NT* serotypes included in denominator (yes/no)
Hill PC.,(2006) [19] data in [21]	Gambia	PCV7+6A	<5 y	108/197 (55%)	226/617 (37%)	186/674 (28%)	Yes
Adetifa IM., et al. (2012 [34]	Nigeria	PCV7	<5 y	173/375 (46%)	19/63(30%)	39/90 (43%)	Yes
Adetifa IM., et al. (2012) [34]	Nigeria	PCV10	<5 y	174/375 (46%)	19/63(30%)	41/90 (45%)	Yes
Adetifa IM., et al. (2012) [34]	Nigeria	PCV13	<5 y	264/375 (70%)	27/63(43%)	52/90 (58%)	Yes
Darboe et al (2012) [39] *, data in [58]	Gambia	PCV13	<1 y	97/143 (68%)		6/26 (23%)	No
Mueller et al. (2012) [49]	Burkina Faso	PCV13	<5 y	45/80 (56%)	20/57 (35%)	9/28 (32%)	No
Turner et al. (2012) [56] *	Thailand^1^	PCV13	<2 y	105/188 (56%)		16/57 (28%)	No
van Gils E. et al (2009) [57] *	Netherlands	PCV7	<2 y	115/213 (54%)		27/67 (40%)	Yes
Reis JN. et al. (2008) [54]	Brazil	PCV7	<5 y	12/33 (36%)	12/43 (28%)	7/19 (37%)	Yes
Hammitt et al (2006) [3]	USA (Alaska)	PCV7	<5 y	209/377 (55%)		78/275 (28%)	Yes
Regev-Yochay (2012) [52]	Occup. Palest Terr.^2^	PCV7	<5 y	65/189 (34%)		6/30 (20%)	Yes
Regev-Yochay (2012) [52]	Occup. Palest Terr.	PCV10	<5 y	69/189 (37%)		6/30 (20%)	Yes
Regev-Yochay (2012) [52]	Occup. Palest Terr.	PCV13	<5 y	93/189 (49%)		9/30 (30%)	Yes
Regev-Yochay e(2004) [16] *	Israel	PCV7	<5 y	87/200 (44%)		8/29 (28%)	No
Hussain (2005) [18] data in [20]	UK	PCV7	<5 y	57/87 (66%)	8/15 (53%)	9/18 (50%)	No
Hussain (2005) [18] data in [20]	UK	PCV13	<5 y	72/87 (83%)	11/15 (73%)	10/18 (55%)	No

1 Maela refugee camp at the Thailand-Myanmar border.

2 Gaza strip.

* data marked with (*) are based on approximation. See the Supporting Information section for further details.

**Figure 3:**
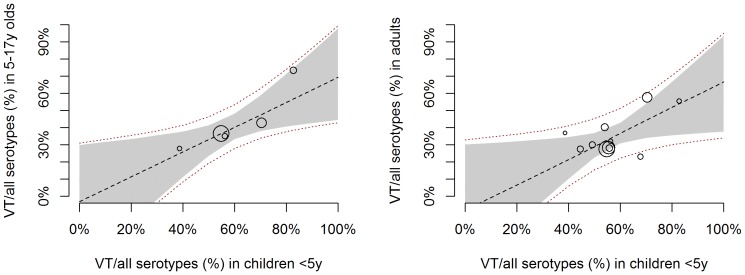
VT proportion in carried serotypes among <5 y and older ages: scatter plot and fitted model. Each circle corresponds to one study, with the circle size proportional to the study size (i.e. number of individuals contributing to the x and y axis estimates). The lines correspond to the fitted Bayesian linear meta-regression model. The dashed black line shows the median posterior estimate and the grey shaded area the 95% credible interval around the median. The red dotted lines represent the 95% prediction interval.

We found a positive linear relationship between the proportion of VT serotypes isolated from carriers in children under five and that in 5–17 y olds or in adults (Figure 3). The intercept was centred around zero in both models, and the slope of the coefficient was 0.73 (Credible intervals (CrI) 0.10–1.37) in 5–17 y old and 0.73 (95%CrI 0.06; 1.32) in adult carriers compared to the proportion of VT serotypes in children <5 y. Further details on the model coefficients are provided in Table 3.

**Figure 4:**
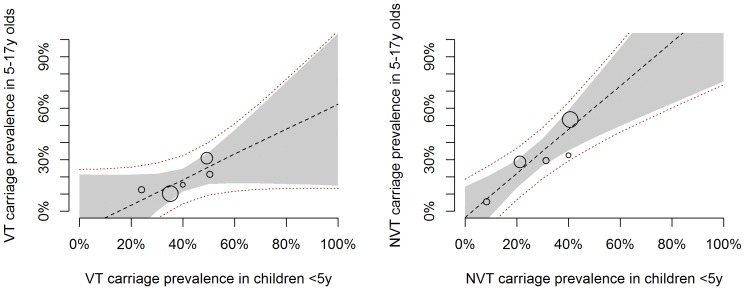
VT and NVT prevalence in 5–17 y olds against <5 y olds: scatter plot and fitted model. Each circle corresponds to one study, with the circle size proportional to the study size (i.e. number of individuals contributing to the x and y axis estimates). The lines correspond to the fitted Bayesian linear meta-regression model. The dashed black line shows the median posterior estimate and the grey shaded area the 95% credible interval around the median. The red dotted lines represent the 95% prediction interval.

**Figure 5:**
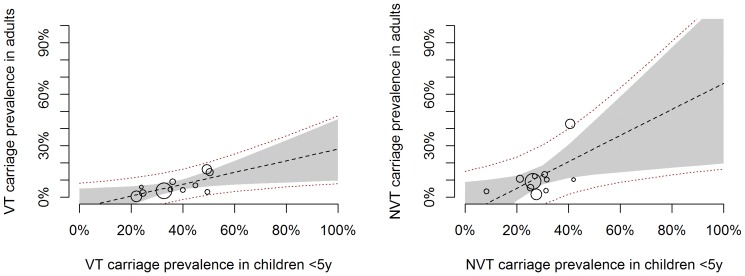
VT and NVT prevalence in adults against <5 y olds: scatter plot and fitted model. Each circle corresponds to one study, with the circle size proportional to the study size (i.e. number of individuals contributing to the x and y axis estimates). The lines correspond to the fitted Bayesian linear meta-regression model. The dashed black line shows the median posterior estimate and the grey shaded area the 95% credible interval around the median. The red dotted lines represent the 95% prediction interval.

We then analysed the relationship between VT and NVT carriage prevalence in <5 y olds and 5–17 y olds or adults. As for the overall carriage prevalence, there was good evidence of a linear trend, with the prevalence of both VT and NVT in older age groups increasing with increasing VT and NVT prevalence in <5 s (Figures 4 and 5). However, given the shift in VT/NVT distribution in older age groups, the prevalence of VT serotypes in 5–17 y olds and adults compared to that in children under five was comparatively lower than that of NVT serotypes (Table 2).

We performed subgroup analyses for VT serotypes included in PCV7 and VT serotypes included in PCV13, and found no difference in the coefficient obtained. Hence for studies reporting estimates for both PCV7 and PCV13 we included in the final analysis estimates with VT serotypes included in the higher valency vaccine.

## Discussion

In this systematic review and meta-analysis the prevalence of *Streptococcus pneumoniae* carriage in the nasopharynx of children aged under five was strongly correlated with the prevalence of nasopharyngeal carriage in older age groups. Furthermore, we found that the proportion of carriage attributed to vaccine serotypes was consistently decreasing with age. Our study provides a tool to help make informed predictions, however with some uncertainty, on the carriage prevalence and serotype distribution in older children and adults solely based on data in children <5 y of age, which are more widely available.

The results of this study showed that despite the important geographic heterogeneity in carriage prevalence, there is a stable linear relationship between the carriage prevalence in young children and that in older children and adults. Such relationship also held for VT and NVT separately, although with different magnitude given the shift in serotype distribution in older age groups, with proportionally more NVT and less VT carriers. While a proportional decrease of carriage prevalence by age, as described by a linear correlation with an intercept centred around zero, did not describe the data well in most instances, the addition of a flexible intercept allowed for a good description of the age-dependent carriage association.

Although the decrease in carriage prevalence through childhood is a well established fact, the between age group correlation in prevalence estimates has – to the best of our knowledge – not been previously described. These results are important to help improve our understanding of carriage and disease dynamics in the population, assess the potential population-wide effects of vaccination programmes and help design appropriate vaccination strategies. Given the high carriage prevalence rates found in children in many developing countries the indirect impact of routine infant PCV immunization on older children and adult populations in such countries is likely to be high, as we find that carriage rates in those age groups are likely to high as well.

The general decrease in prevalence with increasing age can be caused by numerous factors, including the decrease in the duration of carriage with age [22], [23], the reduction in the number of effective contacts as age increases, as well as the general maturation of the immune system [24].

Immunity induced by *S.pneumoniae* carriage is complex and still poorly understood [25]. Although carriage acquisition leads to the development of capsular antibodies, evidence suggests that such antibodies may not be the primary driver of the decrease in duration and prevalence of carriage with age [26]. Mouse models have shown that the development of immunity against colonization in mice depends on CD4+ T cells rather than serotype-specific antibodies as such, in particular T-cells secreting IL-17A (T_H_17 cells) [27], and there is evidence that T_H_17 cells play a role in immunity against carriage in humans too [28].

Yet despite the uncertainty around the exact immune mechanisms, or the contribution of each of those towards acquired long term immunity, epidemiological evidence suggest that serotype-specific immunity against colonization is induced by acquisition of some serotypes such as 6A, 14 and 23F, which are included in PCV formulations and are some of the most prevalent serotypes in early childhood [24]. Hence the progressive acquisition of immunity against VT serotypes may also explain the shift in serotype distribution towards proportionally more NVT as age increases, as immunity against VT serotypes acquired in early childhood may reduce the likelihood of acquiring such serotypes later in life [29].

There are several direct applications of our study results.

Given that most studies are confined to children <5 y of age only, the results of this study are particularly useful in the context of the progressive introduction of PCV10 or PCV13 in many developing countries, in order to help estimate and appraise the possible impact of the vaccine across age groups. For example, nasopharyngeal carriage estimates are central to dynamic models of disease transmission [30], [31], which can be used to model pre-vaccination dynamics and estimate post vaccination trends. With the results of this study, such models could be implemented in settings in which pre-vaccination data are only available in young children.

The quantification of the magnitude of change in carriage prevalence between children <5 y and older age groups is also helpful in the sample size calculations of nasopharyngeal carriage surveys across age groups.

While the specific associations found between VT and NVT carriage prevalence across age groups may not hold for new higher valency vaccines under development, the estimates of overall carriage prevalence across age groups may help evaluate the possible population wide impacts on carriage of new protein-based or killed whole cell serotype-independent candidate vaccines [28].

As a practical application of the presented work we predicted the carriage prevalence in older children and adults based on carriage in under 5 year olds in an aboriginal population in the Northern Territory in Australia [33]. This study was not included in the analysis as it was conducted after the introduction of PCV. However, no change in overall carriage prevalence was observed in the three first years post PCV in this population. Hence we assumed that under a scenario of full serotype replacement overall carriage estimates by age group after PCV implementation would match those from the pre-PCV period. Using such data, we estimated the prevalence in 5–17 y olds and adults to be 58.1% (95% prediction 38.7–77.4%) and 32.4% (95%prediction 14.8–48.8%) respectively, based on data in children under five. This closely matches the study estimates, with the prevalence in 5–17 y olds estimated at 60.9% (95%CI 54.5% - 67.0%) and that in adults estimated at 26.0% (95%CI 22.3–29.9%).

Our study also suffers a range of limitations.

We did not restrict our analysis to any particular design and sampling strategy, and recruitment bias is likely to have occurred, in particular for studies based on convenience rather than random sampling, which was seen frequently for the adult age group. However, our subgroup analyses showed no difference in the prediction estimates of the overall carriage prevalence obtained with studies based on random samples of adults compared to studies based on a convenient sample of relatives or carers of the children included.

By restricting the analysis to broad age groups, we overlooked changes in carriage prevalence within each of those groups. In particular, the prevalence of carriage in the 5–17 y olds is known to decline between the ages of 5 and 17 years. This may also account for some of the heterogeneity seen between studies, as in many studies the age representation of the 5–17 y olds in the study sample may not have matched that of the general population.

Between-study heterogeneity may also have resulted from individual confounding factors associated with carriage prevalence, which we were unable to account for, such as malnutrition, antibiotic use or smoking [32], [33].

Further, although standard WHO laboratory procedures [14] were reported in all studies, differences in swabbing techniques, number of colonies plated, processing of specimens and culture may also account for some of the differences seen. There was however good homogeneity in the serotyping methods used in all studies included.

We could not specifically estimate the carriage prevalence among the elderly as a function of that in young children, given the paucity of data. Having specific estimates in elderly would be important however, given the particularly high burden of pneumococcal disease in that age group and the potential indirect impact of routine PCV on carriage in them. While no significant difference in carriage prevalence between younger and older adults was reported in two studies that provided specific data on older adults or elderly [16], [17], more data are required to enable specific estimates to be made for that age group.

Finally, model estimates of VT and NVT prevalence in older age groups as a function of that in young children were prone to more uncertainty than in the models based on overall carriage prevalence, given that fewer studies reported specific carriage data by groups of serotypes. In addition, those models were based on prevalence estimates which were mostly confined to the lower prevalence levels, resulting in substantial model uncertainty for high prevalence estimates. Further carriage studies will help improve the precision around such estimates, and the analysis can easily be updated with the model code provided in the Supporting Information.

## Conclusions

Information on patterns of nasopharyngeal colonisation in individuals not directly targeted by pneumococcal conjugate vaccination is scarce but plays an important role in the consideration of the indirect impact of PCVs. We here present evidence that a non-trivial stable relationship between child and both adolescent and adult carriage rates holds. Furthermore we show that a similar relationship for the proportion of vaccine type and non-vaccine type carriage is present. We exploit these and provide a tool to make an informed prediction of carriage rates in adolescents and adults based on childhood carriage rates only, including the associated uncertainty. Further carriage studies on broad age ranges will allow narrowing of the prediction intervals. If designed accordingly these could also provide the basis for childhood carriage informed estimates of carriage in the elderly population which is particularly affected by pneumococcal disease.

## Supporting Information

Figure S1 **Overall carriage prevalence in adults against <5 y olds, by sampling characteristics of the adult population: scatter plot and fitted model.** Each circle corresponds to one study, with the circle size proportional to the study size (i.e. number of individuals contributing to the x and y axis estimates). The lines correspond to the fitted Bayesian linear meta-regression model. The dashed black line shows the median posterior estimate and the grey shaded area the 95% credible interval around the median. The red dotted lines represent the 95% prediction interval.
(TIFF)

Checklist S1 **PRISMA checklist.**
(DOC)

File S1 **Details about data extraction from the studies included in the meta-regression analysis.**
(DOC)

File S2 **Model code.** Text file of the R model code.
(TXT)
