# Supplementary figures and images for: Age-Dependent Prevalence of Nasopharyngeal Carriage of Streptococcus pneumoniae before Conjugate Vaccine Introduction: A Prediction Model Based on a Meta-Analysis
Source: PLoS One. 2014 Jan 23;9(1):e86136. doi: 10.1371/journal.pone.0086136 (PMC3900487; doi:10.1371/journal.pone.0086136)

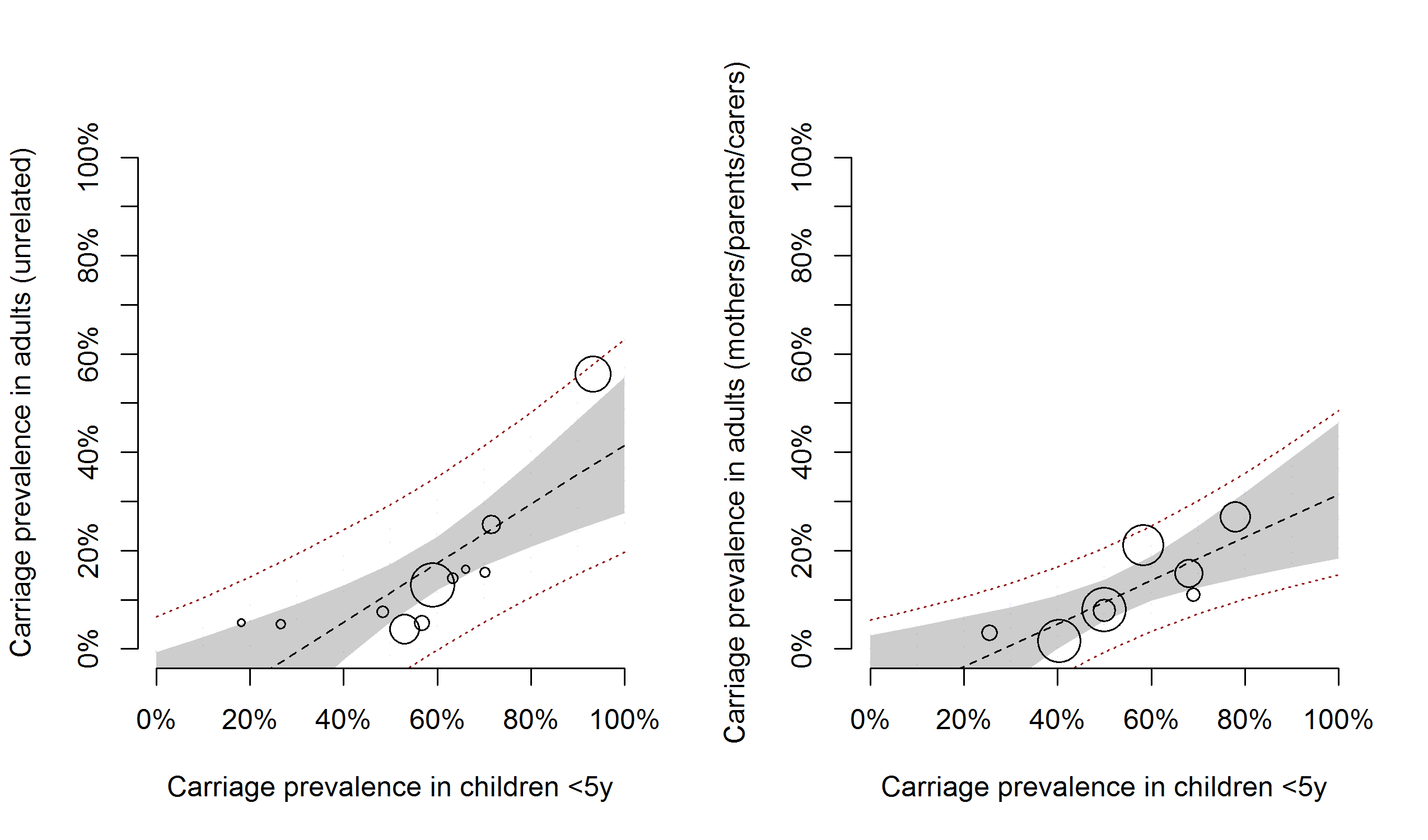

Supplement: Figure S1 — Overall carriage prevalence in adults against <5 y olds, by sampling characteristics of the adult population: scatter plot and fitted model. Each circle corresponds to one study, with the circle size proportional to the study size (i.e. number of individuals contributing to the x and y axis estimates). The lines correspond to the fitted Bayesian linear meta-regression model. The dashed black line shows the median posterior estimate and the grey shaded area the 95% credible interval around the median. The red dotted lines represent the 95% prediction interval. (TIFF) [file pone.0086136.s001.tiff]
