# Supplementary material for: Age-Dependent Prevalence of Nasopharyngeal Carriage of Streptococcus pneumoniae before Conjugate Vaccine Introduction: A Prediction Model Based on a Meta-Analysis
Source: PLoS One. 2014 Jan 23;9(1):e86136. doi: 10.1371/journal.pone.0086136 (PMC3900487; doi:10.1371/journal.pone.0086136)
Supplement: File S1 — Details about data extraction from the studies included in the meta-regression analysis. (DOC) [file pone.0086136.s003.doc]

**File S1: Details about data extraction from the studies included in the meta-regression analysis**

1. Abdullahi et al.

Estimates were directly provided in the paper.

1. Adetifa et al

Overall carriage estimates were obtained based on the reported prevalence by age group and the number of individuals included in each age group. Estimates for PCV7, PCV10 and PCV13 serotypes were provided directly in the paper

1. Bello Gonzales et al.

Estimates were directly provided in the paper.

1. Cekmez et al

Estimates were directly provided in the paper.

1. Chen et al.

Estimates were directly provided in the paper.

1. Dagan et al.

Estimates were directly provided in the paper.

1. Darboe et al.

We used data published in a subsequent study as the latter provided more detailed information on carriage than the original study. A birth cohort was followed up to the age of one year, and nasopharyngeal (NP) samples were taken at 0, 2, 5, and 12 months. We calculated the carriage prevalence as the average prevalence between 0-12 months, based on monthly estimates. The carriage prevalence for months for which no NP sample was taken was calculated as the average of the NP prevalence of the two nearest months with available estimates. Similarly, NP samples from mothers were taken at their child’s birth 2 months, 5 months and 12 months later. We considered the sample taken at birth to be more representative from the carriage prevalence in the general adult population than those taken during the first year, during which the prevalence in mothers increase as a result of close infant-mother interaction. Hence only the carriage prevalence obtained at birth were used for the analysis of the carriage prevalence in adults.

For the calculation of VT and NVT carriage prevalence NT serotypes were excluded from the denominator

1. Dhakal et al.

Carriage estimates were directly provided in the paper

1. Granat et al.

Carriage estimates were directly provided in the paper

1. Greenberg et al.

Carriage estimates were directly provided in the paper

1. Hammitt et al.

Carriage estimates were directly provided in the paper

1. Henriqus Normark et al.

Carriage estimates were directly provided in the paper

1. Hill et al.

Carriage estimates were directly provided in the paper

1. Hussain et al.

Prevalence estimates were based on the study data later reported by Flasche et al , where the prevalence by age group was adjusted for multiple testing. Sample size by age group was estimated based on the prevalence estimates in and the number of cases, by age group.

For the calculation of VT and NVT carriage prevalence NT serotypes were excluded from the denominator

1. Inostroza et al.

The samples were obtained from two cities in two different populations: (1) Santiago, where children with Invasive Pneumococcal Disease (IPD) were sampled as well as their contacts, in addition to a sample of healthy children attending day care centres, and (ii) Temuco, where healthy children attending day care centre and hospital staff were sampled. For analysis, only samples from Temuco were considered.

1. Kaltoft et al.

Carriage estimates were directly provided in the paper

1. Leino et al.

Carriage estimates were directly provided in the paper

1. Lloyd-Evans et al.

The study included index cases <5 years of age admitted to the hospital with a diagnosis of IPD, as well as community based healthy controls <5 years of age. Samples of family members of index cases were obtained on the day of admission as well as one month later. We included data from samples obtained in healthy children <5y in the analysis, as well as data from the samples from family members of cases of IPD obtained one month after the index cases’ hospital admission.

1. Lo et al.

Estimates were directly provided in the paper.

1. Mueller et al.

Overall carriage estimates obtained based on the reported prevalence by age group and the number of individuals included in each age group.

1. Nunes et al

The study participants consisted of 120 pairs of HIV positive mothers and HIV exposed children and 123 HIV unexposed mother-infant pairs. Swabs were collected from infants at five different times (average age of 4m, 7m, 9m, 12m and 16m). The carriage prevalence in children and mothers was approximated by the number of positive swabs in each age group over the total number of swabs collected. This calculation was performed to obtain overall carriage estimates. Estimates for PCV7 and PCV13 serotypes could not be obtained. Only the data from the HIV unexposed mother-infant pairs were used in this analysis.

1. Parry et al.

Estimates were directly provided in the paper.

1. Regev-Yochay et al.

The study population comprised of children and adults attending any of the health care centres in four large cities in Israel for any reason. Of the 404 children included, 173 attended for an upper respiratory tract infection (URTI) and among adults, 192 of the 1300 included had an URTI on the day. For the purpose of the analysis of overall carriage prevalence, children and adults with an URTI were excluded. However in the analysis of VT and NVT prevalence and distribution all data were included as data available from the manuscript did not differentiate between those admitted or not for URTI.

1. Regev-Yochay et al.

Overall carriage estimates obtained based on the reported prevalence by age group and the number of individuals included in each age group.

1. Reichler et al.

Exact numbers were obtained based on a bar chart of the prevalence by age group and the number of individuals in each age group. We included all children <64months in the calculation of the carriage prevalence in <5y olds and children in the after kindergarten/after school aged 5-10y in the older children age groups. Estimates from a group of children aged 3-7y who attended a Montessori were not included as the age band stretched over the 2 age groups.

1. Reis et al.

Carriage estimates were directly provided in the paper

1. Sener et al.

Carriage estimates were directly provided in the paper

1. Turner et al.

A cohort of newborns and their mothers were followed from birth to 24 months of age, and monthly swabs were taken. After a rapid increase in NP carriage prevalence in the first 2 months the prevalence of carriage in children remained stable between the ages of 3 and 24 months. The carriage prevalence was also stable in mothers. We calculated the carriage prevalence as the total number of positive swabs out of all swabs taken (children: 3363/4191 and mothers 1033/4195), and translated this in number of cases based on the sample size of children (n=234) and mothers (n=231) and the prevalence estimates. Given the stability of the positivity rate by monthly age group, we assumed for the analysis that the prevalence in <1y was equivalent to that in <2y.

Similarly, for VT and NVT serotypes, the calculation was based on the average VT and NVT positive swabs (excluding NT) and the numbers of carriers calculated in each age group.

1. van Gils et al.

An individual RCT was conducted. We used carriage estimates from the control group only (i.e. unvaccinated). Children were swabbed at 12 months, 18 months and 24 months. For carriage estimates in infants (<1y) we only included estimates at 12 months of age. However, for the VT and NVT carriage prevalence we calculated the average over the three sampling periods, at 12 months, 18 months and 24 months. In adults swabs were taken from parents when their children were 12 months and 24 months old. We calculated the carriage prevalence based on the average of samples taken at 12 months and samples taken at 24 months.

**REFERENCES**

1. Abdullahi O, Nyiro J, Lewa P, Slack M, Scott JA (2008) The descriptive epidemiology of Streptococcus pneumoniae and Haemophilus influenzae nasopharyngeal carriage in children and adults in Kilifi district, Kenya. Pediatr Infect Dis J 27: 59-64.

2. Adetifa IM, Antonio M, Okoromah CA, Ebruke C, Inem V, et al. (2012) Pre-vaccination nasopharyngeal pneumococcal carriage in a Nigerian population: epidemiology and population biology. PLoS One 7: e30548.

3. Bello Gonzalez T, Rivera-Olivero IA, Pocaterra L, Spadola E, Araque M, et al. (2010) [Pneumococcal carriage in mothers and children of the Panare Amerindians from the State of Bolivar, Venezuela]. Rev Argent Microbiol 42: 30-34.

4. Cekmez F, Karademir F, Tanju IA, Meral C, Pirgon O, et al. (2009) Pneumococcal serotypes recovered from health children and their possible association with risk factor in istanbul, Turkey. Int J Biomed Sci 5: 101-104.

5. Chen CJ, Huang YC, Su LH, Lin TY (2007) Nasal carriage of Streptococcus pneumoniae in healthy children and adults in northern Taiwan. Diagn Microbiol Infect Dis 59: 265-269.

6. Dagan R, Gradstein S, Belmaker I, Porat N, Siton Y, et al. (2000) An outbreak of Streptococcus pneumoniae serotype 1 in a closed community in southern Israel. Clin Infect Dis 30: 319-321.

7. Darboe MK, Thurnham DI, Morgan G, Adegbola RA, Secka O, et al. (2007) Effectiveness of an early supplementation scheme of high-dose vitamin A versus standard WHO protocol in Gambian mothers and infants: a randomised controlled trial. Lancet 369: 2088-2096.

8. Darboe MK, Fulford AJ, Secka O, Prentice AM (2010) The dynamics of nasopharyngeal streptococcus pneumoniae carriage among rural Gambian mother-infant pairs. BMC Infect Dis 10: 195.

9. Dhakal R, Sujatha S, Parija SC, Bhat BV (2010) Asymptomatic colonization of upper respiratory tract by potential bacterial pathogens. Indian J Pediatr 77: 775-778.

10. Granat SM, Mia Z, Ollgren J, Herva E, Das M, et al. (2007) Longitudinal study on pneumococcal carriage during the first year of life in Bangladesh. PediatrInfectDisJ 26: 319-324.

11. Greenberg D, Broides A, Blancovich I, Peled N, Givon-Lavi N, et al. (2004) Relative importance of nasopharyngeal versus oropharyngeal sampling for isolation of Streptococcus pneumoniae and Haemophilus influenzae from healthy and sick individuals varies with age. J Clin Microbiol 42: 4604-4609.

12. Hammitt LL, Bruden DL, Butler JC, Baggett HC, Hurlburt DA, et al. (2006) Indirect effect of conjugate vaccine on adult carriage of Streptococcus pneumoniae: an explanation of trends in invasive pneumococcal disease. JInfectDis 193: 1487-1494.

13. Henriqus Normark B, Christensson B, Sandgren A, Noreen B, Sylvan S, et al. (2003) Clonal analysis of Streptococcus pneumoniae nonsusceptible to penicillin at day-care centers with index cases, in a region with low incidence of resistance: emergence of an invasive type 35B clone among carriers. Microb Drug Resist 9: 337-344.

14. Hill PC, Akisanya A, Sankareh K, Cheung YB, Saaka M, et al. (2006) Nasopharyngeal carriage of Streptococcus pneumoniae in Gambian villagers. Clin Infect Dis 43: 673-679.

15. Hussain M, Melegaro A, Pebody RG, George R, Edmunds WJ, et al. (2005) A longitudinal household study of Streptococcus pneumoniae nasopharyngeal carriage in a UK setting. EpidemiolInfect 133: 891-898.

16. Flasche S, van Hoek AJ, Sheasby E, Waight P, Andrews N, et al. (2011) Effect of pneumococcal conjugate vaccination on serotype-specific carriage and invasive disease in England: a cross-sectional study. PLoSMed 8: e1001017.

17. Inostroza J, Trucco O, Prado V, Vinet AM, Retamal G, et al. (1998) Capsular serotype and antibiotic resistance of Streptococcus pneumoniae isolates in two Chilean cities. Clin Diagn Lab Immunol 5: 176-180.

18. Kaltoft MS, Skov Sorensen UB, Slotved HC, Konradsen HB (2008) An easy method for detection of nasopharyngeal carriage of multiple Streptococcus pneumoniae serotypes. J Microbiol Methods 75: 540-544.

19. Leino T, Hoti F, Syrjanen R, Tanskanen A, Auranen K (2008) Clustering of serotypes in a longitudinal study of Streptococcus pneumoniae carriage in three day care centres. BMC Infect Dis 8: 173.

20. Lloyd-Evans N, O'Dempsey TJ, Baldeh I, Secka O, Demba E, et al. (1996) Nasopharyngeal carriage of pneumococci in Gambian children and in their families. Pediatr Infect Dis J 15: 866-871.

21. Lo WT, Wang CC, Yu CM, Chu ML (2003) Rate of nasopharyngeal carriage, antimicrobial resistance and serotype of Streptococcus pneumoniae among children in northern Taiwan. J Microbiol Immunol Infect 36: 175-181.

22. Mueller JE, Yaro S, Ouedraogo MS, Levina N, Njanpop-Lafourcade BM, et al. (2012) Pneumococci in the African meningitis belt: meningitis incidence and carriage prevalence in children and adults. PLoS One 7: e52464.

23. Nunes MC, Shiri T, van Niekerk N, Cutland CL, Groome MJ, et al. (2013) Acquisition of Streptococcus pneumoniae in pneumococcal conjugate vaccine-naive South African children and their mothers. Pediatr Infect Dis J 32: e192-205.

24. Parry CM, Diep TS, Wain J, Hoa NT, Gainsborough M, et al. (2000) Nasal carriage in Vietnamese children of Streptococcus pneumoniae resistant to multiple antimicrobial agents. Antimicrob Agents Chemother 44: 484-488.

25. Regev-Yochay G, Raz M, Dagan R, Porat N, Shainberg B, et al. (2004) Nasopharyngeal carriage of Streptococcus pneumoniae by adults and children in community and family settings. Clin Infect Dis 38: 632-639.

26. Regev-Yochay G, Abullaish I, Malley R, Shainberg B, Varon M, et al. (2012) Streptococcus pneumoniae carriage in the Gaza strip. PLoS One 7: e35061.

27. Reichler MR, Allphin AA, Breiman RF, Schreiber JR, Arnold JE, et al. (1992) The spread of multiply resistant Streptococcus pneumoniae at a day care center in Ohio. J Infect Dis 166: 1346-1353.

28. Reis JN, Palma T, Ribeiro GS, Pinheiro RM, Ribeiro CT, et al. (2008) Transmission of Streptococcus pneumoniae in an urban slum community. J Infect 57: 204-213.

29. Sener B, Arikan S, Ergin MA, Gunalp A (1998) Rate of carriage, serotype distribution and penicillin resistance of Streptococcus pneumoniae in healthy children. Zentralbl Bakteriol 288: 421-428.

30. Turner P, Turner C, Jankhot A, Helen N, Lee SJ, et al. (2012) A longitudinal study of Streptococcus pneumoniae carriage in a cohort of infants and their mothers on the Thailand-Myanmar border. PLoSOne 7: e38271.

31. van Gils EJ, Veenhoven RH, Hak E, Rodenburg GD, Bogaert D, et al. (2009) Effect of reduced-dose schedules with 7-valent pneumococcal conjugate vaccine on nasopharyngeal pneumococcal carriage in children: a randomized controlled trial. JAMA 302: 159-167.
